# Supplementary material for: Retinoblastoma from human stem cell-derived retinal organoids
Source: Nat Commun. 2021 Jul 27;12:4535. doi: 10.1038/s41467-021-24781-7 (PMC8316454; doi:10.1038/s41467-021-24781-7)
Supplement: Supplementary file 1 — Supplementary Information [file 41467_2021_24781_MOESM1_ESM.pdf]

Supplementary Information

Retinoblastoma from Human Stem Cell-Derived Retinal Organoids

Jackie L. Norrie, Anjana Nityanandam, Karen Lai, Xiang Chen, Matthew Wilson, Elizabeth Stewart, Lyra Griffiths, Hongjian Jin, Gang Wu, Brent Orr, Quynh Tran, Sariah Allen, Colleen Reilly, Xin Zhou, Jiakun Zhang, Kyle Newman, Dianna Johnson, Rachel Brennan and Michael A. Dyer

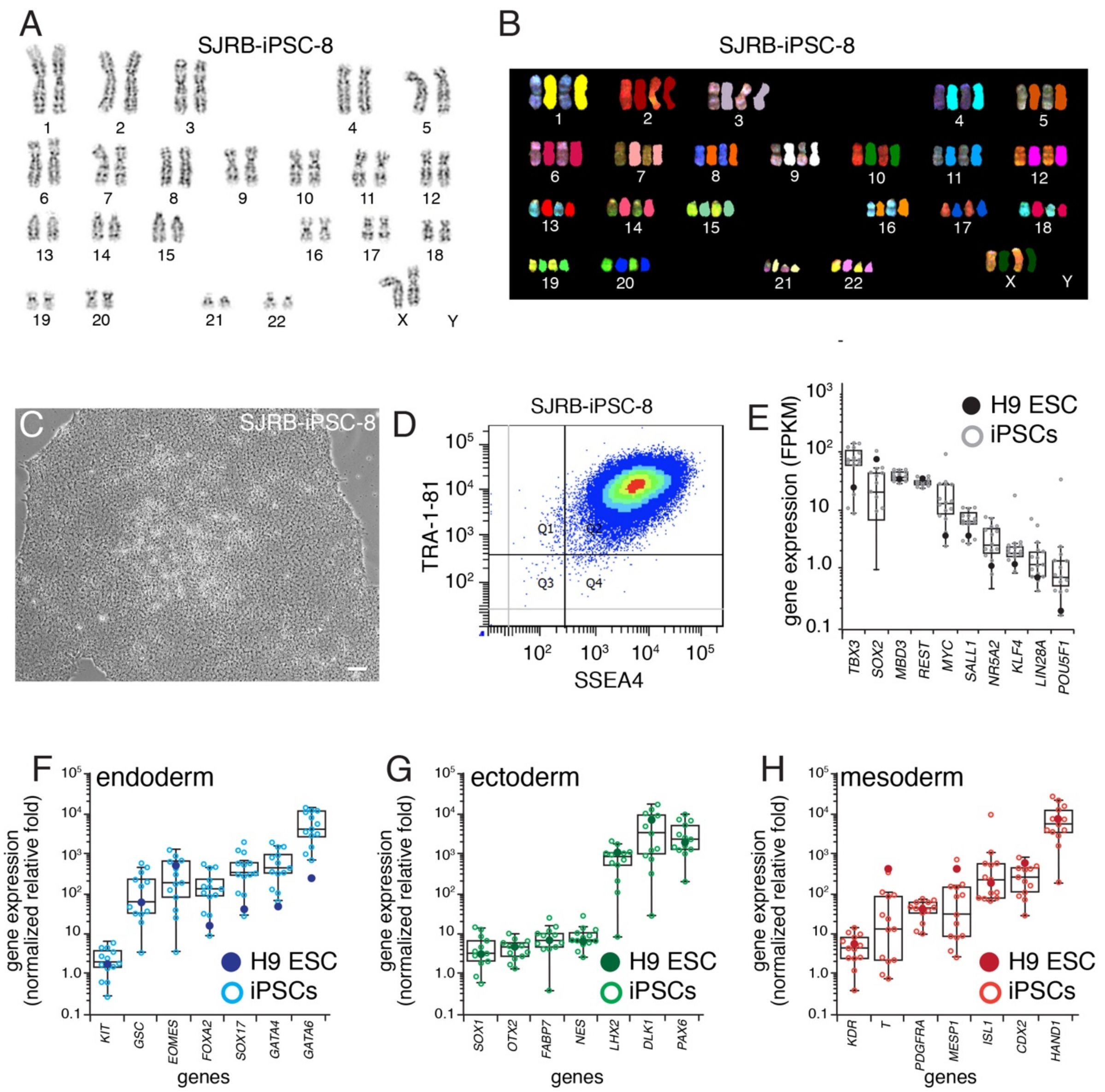
A
SJRB-iPSC-8
B
SJRB-iPSC-8
C
SJRB-iPSC-8
D
SJRB-iPSC-8
TRA-1-81
SSEA4
Q1
Q2
Q3
Q4
E
H9 ESC
iPSCs
gene expression (FPKM)
TBX3
SOX2
MBD3
REST
MYC
SALL1
NR5A2
KLF4
LIN28A
POU5F1
F
endoderm
gene expression (normalized relative fold)
KIT
GSC
EOMES
FOXA2
SOX17
GATA4
GATA6
genes
H9 ESC
iPSCs
G
ectoderm
SOX1
OTX2
FABP7
NES
LHX2
DLK1
PAX6
genes
H
mesoderm
KDR
T
PDGFRA
MESP1
ISL1
CDX2
HAND1
genes

Supplementary Figure 1. Characterization of iPSC lines from patients with germline *RB1* mutations. A) Micrograph of representative karyotype for one of the iPSC clones. **B)** Spectral karyotype (SKY) of the same clone shown in (A). **C)** Brightfield micrograph of an iPSC colony. All 15 iPSC lines were analyzed with similar results. **D)** Scatterplot of FACS analysis for two markers of pluripotent cells (Q2 = 99.4% of single cells). **E)** Box and whisker plot of pluripotency gene expression from RNA-seq for each of the 15 individual iPSC lines (open gray circles) and H9 ESCs as a control (filled black circle). **F-H)** Box and whisker plot of endoderm, ectoderm and mesodermal cells from the trilineage assay for each of the 15 individual iPSC lines (open circles) and H9 ESCs (closed circle). Data are normalized relative fold change compared to undifferentiated H9 ESCs. Box and whisker plots include center line as median, box as Q1 and Q3, and whiskers as 1.5x interquartile range. Scale bar, 50 mm.

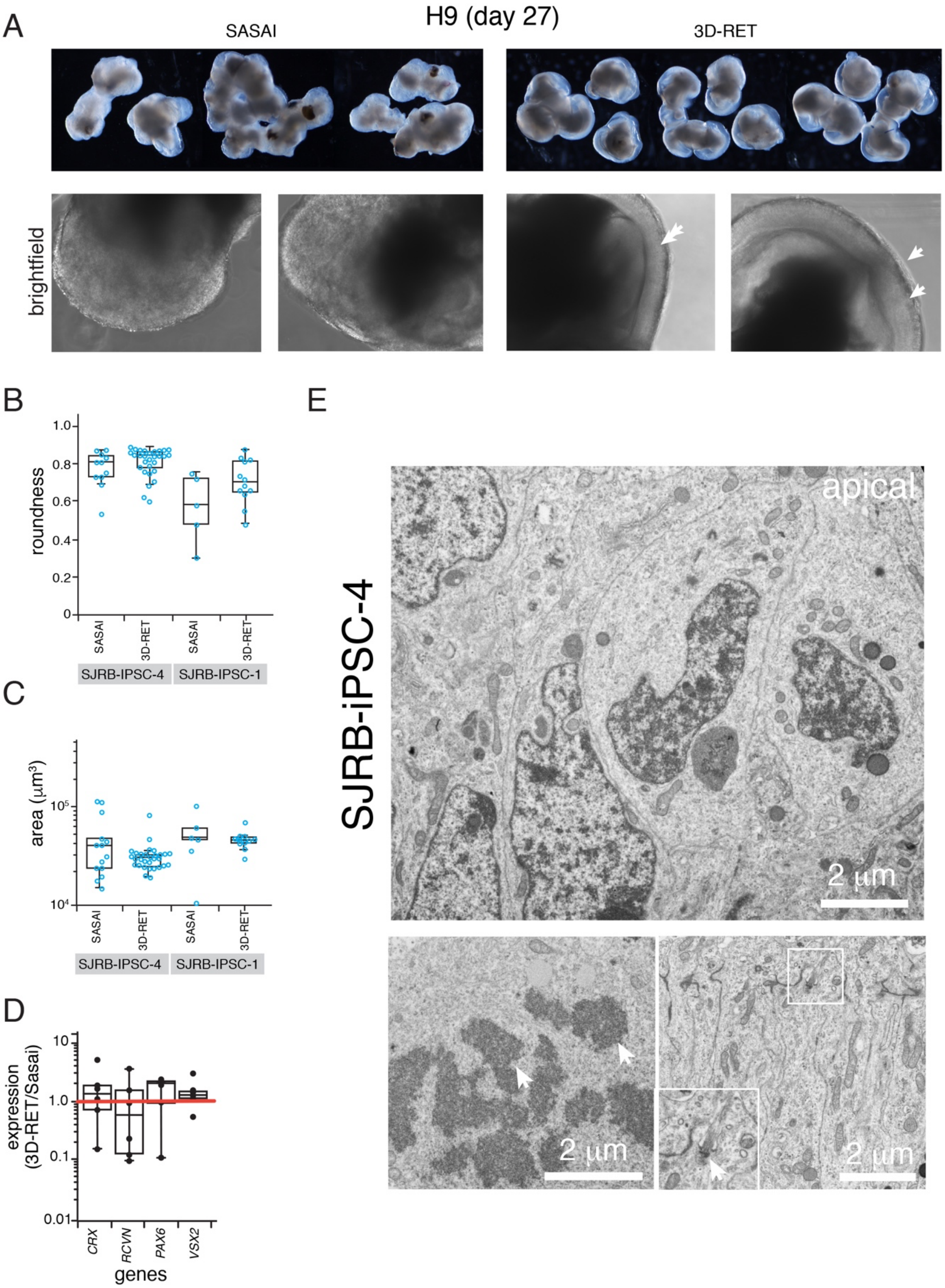
A
H9 (day 27)
SASAI
3D-RET
brightfield
B
roundness
SASAI
3D-RET
SASAI
3D-RET
SJRB-IPSC-4
SJRB-IPSC-1
C
area (μm³)
SASAI
3D-RET
SASAI
3D-RET
SJRB-IPSC-4
SJRB-IPSC-1
D
expression
(3D-RET/Sasai)
CRX
RCVN
PAX6
VSX2
genes
E
SJRB-iPSC-4
apical
2 μm
2 μm
2 μm

Supplementary Figure 2. Characterization of retinal organoids from using two different protocols. A) Micrograph of all the retinal organoids from a single 96 well dish for the same cells side-by-side using the Sasai and 3D-RET protocol. Lower panels show brightfield images with retinal structures (arrows). **B,C)** Box and whisker plot of quantitation of roundness and cross-sectional area of retinal organoids using the two methods. All organoids that were generated from a 96 well dish during a single round of differentiation were quantified. **D)** Box and whisker plot of normalized relative fold expression of retinal genes for iPSC organoids using the two methods (n=6). **E)** Representative electron micrograph of a retinal organoid showing tightly packed cells in the apical surface of the organoid as well as mitotic figure (inset and arrows) and cilium and apical junctions (inset and arrow). All 15 lines were analyzed with similar results. Box and whisker plots include center line as median, box as Q1 and Q3, and whiskers as 1.5x interquartile range.

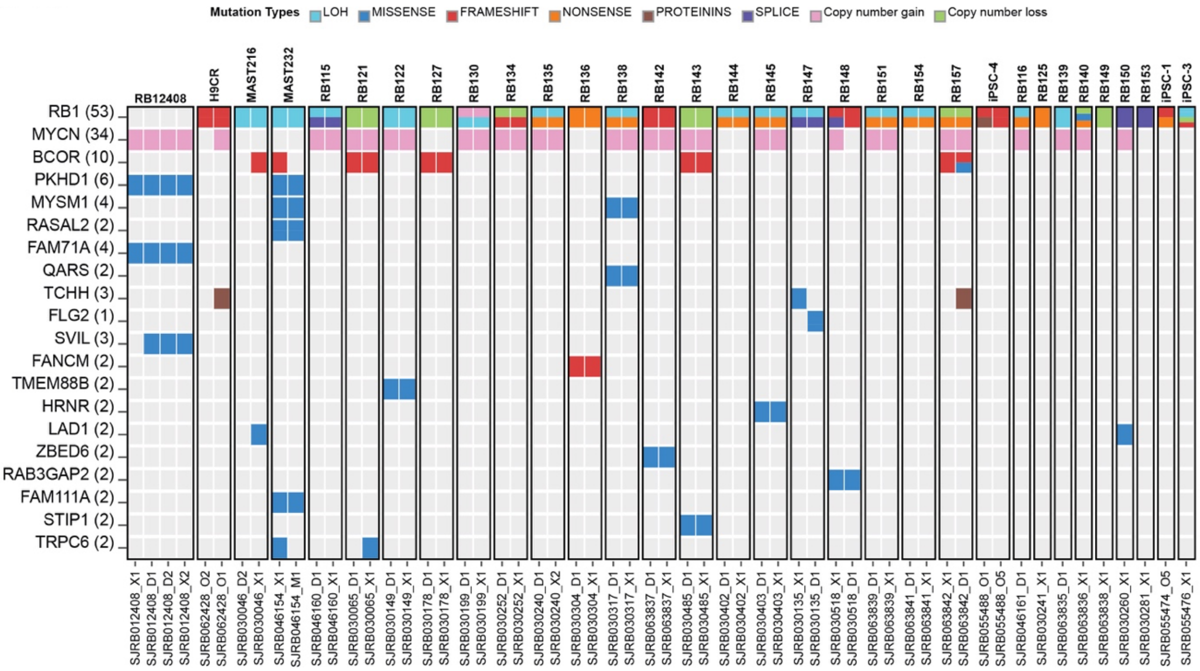
Mutation Types
LOH
MISSENSE
FRAMESHIFT
NONSENSE
PROTEININS
SPLICE
Copy number gain
Copy number loss
RB12408
H9CR
MAST216
MAST232
RB115
RB121
RB122
RB127
RB130
RB134
RB135
RB136
RB138
RB142
RB143
RB144
RB145
RB147
RB148
RB151
RB154
RB157
iPSC-4
RB116
RB125
RB139
RB140
RB149
RB150
RB153
iPSC-1
iPSC-3
RB1 (53)
MYCN (34)
BCOR (10)
PKHD1 (6)
MYSM1 (4)
RASAL2 (2)
FAM71A (4)
QARS (2)
TCHH (3)
FLG2 (1)
SVIL (3)
FANCM (2)
TMEM88B (2)
HRNR (2)
LAD1 (2)
ZBED6 (2)
RAB3GAP2 (2)
FAM111A (2)
STIP1 (2)
TRPC6 (2)
SJRB012408_X1
SJRB012408_D1
SJRB012408_D2
SJRB012408_X2
SJRB062428_O2
SJRB062428_O1
SJRB030046_D2
SJRB030046_X1
SJRB046154_X1
SJRB046154_M1
SJRB046160_D1
SJRB046160_X1
SJRB030065_D1
SJRB030065_X1
SJRB030149_D1
SJRB030149_X1
SJRB030178_D1
SJRB030178_X1
SJRB030199_D1
SJRB030199_X1
SJRB030252_D1
SJRB030252_X1
SJRB030240_D1
SJRB030240_X2
SJRB030304_D1
SJRB030304_X1
SJRB030317_D1
SJRB030317_X1
SJRB063837_D1
SJRB063837_X1
SJRB030485_D1
SJRB030485_X1
SJRB030402_D1
SJRB030402_X1
SJRB030403_D1
SJRB030403_X1
SJRB030135_X1
SJRB030135_D1
SJRB030518_X1
SJRB030518_D1
SJRB063839_D1
SJRB063839_X1
SJRB063841_D1
SJRB063841_X1
SJRB063842_X1
SJRB063842_D1
SJRB055488_O1
SJRB055488_O5
SJRB046161_D1
SJRB030241_X1
SJRB063835_D1
SJRB063836_X1
SJRB063838_X1
SJRB030260_X1
SJRB030281_X1
SJRB055474_O5
SJRB055476_X1

Supplementary Figure 3. Germline and somatic mutations in retinoblastoma. Heatmap of germline and somatic mutations in the retinoblastoma cohort analyzed here. Patient tumors have an _D1 or _D2 designation. O-PDX tumors have an _X1 or _X2 designation. 3D stem cell organoid derived tumors have an _O1, _O2 or _O5 designation.

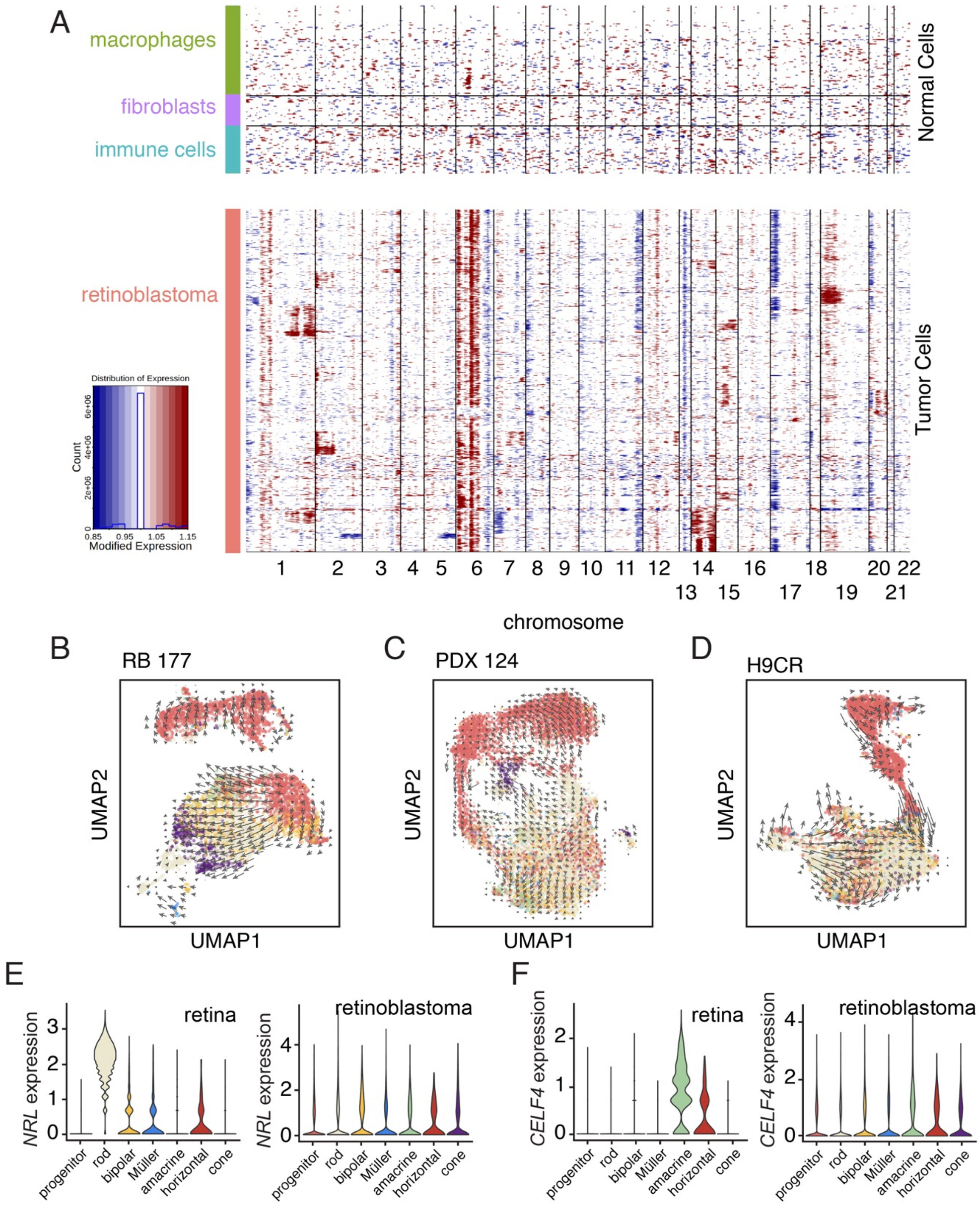

A
macrophages
fibroblasts
immune cells
Normal Cells
retinoblastoma
Tumor Cells
Distribution of Expression
Count
Modified Expression
chromosome
B
RB 177
C
PDX 124
D
H9CR
UMAP1
UMAP2
E
NRL expression
retina
retinoblastoma
progenitor
rod
bipolar
Müller
amacrine
horizontal
cone
F
CELF4 expression

Supplementary Figure 4. Inferred copy number variation in scRNA-seq and RNA velocity analysis. A) Tumor cells can be distinguished from normal cells by inferring the chromosomal copy number of large chromosomal regions from gene expression levels as indicated. Normal cells including macrophages, fibroblasts and immune cells were identified by gene expression signatures of those cell types and the absence of inferred chromosomal copy number changes. **B-D)** RNA velocity analysis of a representative retinoblastoma patient tumor (RB177), O-PDX (PDX 124) and organoid derived tumor (H9CR) with cell identity from the label transfer shown as in Fig. 5. **E,F)** Velocity plot for a rod gene (NRL) and an amacrine/horizontal cell gene (CELF4) for normal retina and retinoblastoma showing that the tumor cells are a hybrid of multiple gene expression programs.

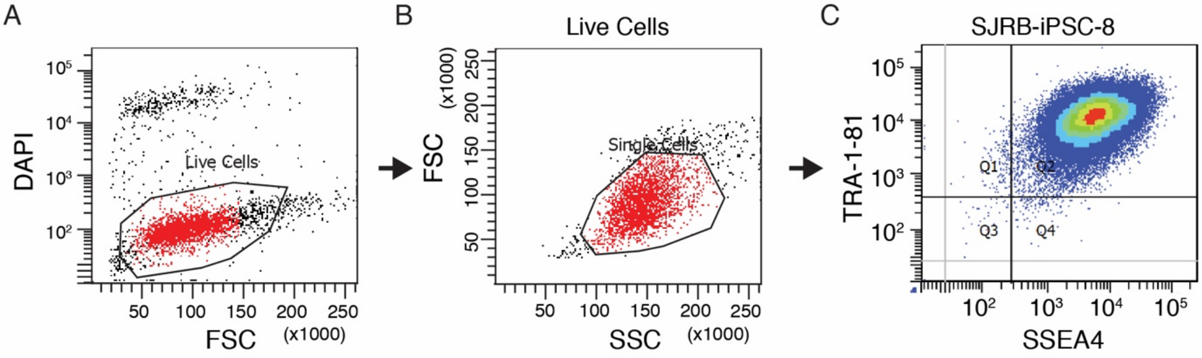

D

Population	#Events	%Parent	%Total
All Events	70,408	####	100.0
Live Cells	56,933	80.9	80.9
Single Cells	50,000	87.8	71.0
Q1	175	0.4	0.2
Q2	49,699	99.4	70.6
Q3	31	0.1	0.0
Q4	95	0.2	0.1

Supplementary Figure 5. Gating Strategy for Flow Cytometry. A) DAPI negative cells were selected as live cells. **B)** Single cells were selected from the population of live cells. **C)** Populations were analyzed for TRA-1-81 and SSEA4 positive cells. **D)** Population statistic for each stage of gating.

Supplementary Table 1. RB1 genotyping primers

Exon	Sequence
Exon 4	GTAGTGATTTGATGTAGAGC
	CCCAGAATCTAATTGTGAAC
Exon 8	AGTAGTAGAATGTTACCAAG
	TACTGCAAAAGAGTTAGCAC
Exon 10	TCTTTAATGAAATCTGTGCC
	GATATCTAAAGGTCACTAAG
Exon 14	GTGATTTTCTAAAATAGCAGG
	TGCCTTGACCTCCTGATCTG
Exon 21	GGTATTTTTAAGACAAAACCATG
	AAGGTCAGACAGAATATATGATCTC
Exon 25	GGTTGCTAACTATGAAACAC
	AGAAATTGGTATAAGCCAGG

Supplementary Table 2. Antibody List

Antibody	Vendor	Cat#	Source	Dilution
PAX6	DSHB	AB_528427	Mouse	1:100
Recoverin	Millipore	AB5585	Rabbit	1:5000
VSX2	Exalpha Biologics Inc.	X1180P	Sheep	1:200
OTX2	Santa Cruz	sc-30659	Goat	1:200
OCT3/4	BD Biosciences	BD 611202	Mouse	1:500

Supplementary Table 3. QRT-PCR primers

Gene	Sequence
PAX6	CTAGCCAGGTTGCGAAGAAC
	GGGCAATCGGTGGTAGTAAA
VSX2	GGCTCCCTGGCTTCTACAC
	ACATTTTTCGATCGCTGGAG
CRX	GCCCCACTATTCTGTCAACG
	CTTCAGAGCCACCTCCTCAC
GAPDH	CCAGCAAGAGCACAAGAGGAA
	GCCCCTCCCCTCTTCAAG
RCVN	ACCAACCAGAAGCTGGAGTG
	CGTGTTTTCATCGTCTGGAA

Supplementary Table 4. RB1 genotyping primers

Assay	Gene	Taqman Probe
Neural	PAX6	Hs00240871_m1
Neural	NEUROG1	Hs01029249_s1
Neural	NEUROG2	Hs00702774_s1
Neural	EMX2	Hs00244574_m1
Neural	FOXG1	Hs01850784_s1
Neural	ASCL1	Hs04187546_g1
Neural	GBX2	Hs00230965_m1
Neural	DLX2	Hs00269993_m1
Neural	DMBX1	Hs00542612_m1
Neural	GAPDH	Hs99999905_m1
Neural	SOX10	Hs00366918_m1
Neural	ATOH1	Hs00245453_s1
Neural	OTX2	Hs00222238_m1
Neural	ZIC1	Hs00602749_m1
Neural	SOX17	Hs00751752_s1
Neural	GATA4	Hs00171403_m1
Neural	FOXA2	Hs00232764_m1
Neural	ETV2	Hs01012850_g1
Neural	MESP1	Hs01001283_g1
Neural	CD34	Hs02576480_m1
Neural	NANOGP1;NANOG	Hs04399610_g1
Retinoblastoma	MYC	Hs00153408_m1
Retinoblastoma	KLF4	Hs00358836_m1
Retinoblastoma	POU5F1	Hs04260367_gH
Retinoblastoma	BMP2	Hs00154192_m1
Retinoblastoma	EPHA7	Hs01033006_m1
Retinoblastoma	GAPDH	Hs99999905_m1
Retinoblastoma	SYK	Hs00895377_m1
Retinoblastoma	RAX	Hs00429459_m1
Retinoblastoma	PDE6H	Hs01124155_m1
Retinoblastoma	RD3	Hs01650935_m1
Retinoblastoma	SIX6	Hs00201310_m1

Supplementary Table 4 continued. RB1 genotyping primers

Assay	Gene	Taqman Probe
Trilineage	18S	Hs99999901_s1
Trilineage	PAX6	Hs01088114_m1
Trilineage	KDR	Hs00911700_m1
Trilineage	KIT	Hs00174029_m1
Trilineage	NES	Hs04187831_g1
Trilineage	PDGFRA	Hs00998018_m1
Trilineage	GSC	Hs00418279_m1
Trilineage	OTX2	Hs00222238_m1
Trilineage	CDX2	Hs01078080_m1
Trilineage	FOXA2	Hs05036278_s1
Trilineage	FABP7	Hs00361424_g1
Trilineage	HAND1	Hs02330376_s1
Trilineage	SOX17	Hs00751752_s1
Trilineage	DLK1	Hs00171584_m1
Trilineage	MESP1	Hs00251489_m1
Trilineage	GATA4	Hs00171403_m1
Trilineage	LHX2	Hs00180351_m1
Trilineage	ISL1	Hs00158126_m1
Trilineage	EOMES	Hs00172872_m1
Trilineage	SOX1	Hs01057642_s1
Trilineage	T	Hs00610080_m1
Trilineage	GATA6	Hs00232018_m1

Supplementary Table 5. RB1 gRNA sequences

Name	gRNA Sequence	Location
SM27.RB1.g2	TGACATAGCATTATCAACTTNGG	chr13:48345124-48345146
SM27.RB1.g3	AGCATTATCAACTTTGGTACNGG	chr13:48345118-48345140
