# Supplementary material for: Retinoblastoma from human stem cell-derived retinal organoids
Source: Nat Commun. 2021 Jul 27;12:4535. doi: 10.1038/s41467-021-24781-7 (PMC8316454; doi:10.1038/s41467-021-24781-7)
Supplement: Supplementary file 3 — Description of Additional Supplementary Files [file 41467_2021_24781_MOESM3_ESM.pdf]

Supplementary Data Legends

File Name: Supplementary Data 1
Description: Patient-derived iPSC line details.

File Name: Supplementary Data 2
Description: Validation and characterization of patient derived iPSCs. The overall characterization details for each iPSC line and clone and RNA-sequencing of each iPSC line, qRT-PCR of the trilineage analysis and neural differentiation assays, and RNA-sequencings of each iPSC line as a retinal organoid at day 45 of retinal differentiation.

File Name: Supplementary Data 3
Description: Single nucleotide variants, structural variants, and indels identified from whole genome sequence analysis of iPSC clones and patient germline.

File Name: Supplementary Data 4
Description: Growth of retinoblastoma from retinal organoids. Germline RB1 mutations in patient tumor and iPSC lines, somatic mutations in organoid derived tumors and reference retinoblastomas. QRT-PCR and RNA-sequencing of retinal organoid derived tumors.

File Name: Supplementary Data 5
Description: Differential DNA methylation between retinal organoids and retinoblastoma.

File Name: Supplementary Data 6
Description: Overall and individual summary of cell type distribution for single cell RNA-sequencing data.
