# Supplementary material for: Retinoblastoma from human stem cell-derived retinal organoids
Source: Nat Commun. 2021 Jul 27;12:4535. doi: 10.1038/s41467-021-24781-7 (PMC8316454; doi:10.1038/s41467-021-24781-7)
Supplement: Supplementary file 10 — Reporting Summary [file 41467_2021_24781_MOESM10_ESM.pdf]

nature research

Corresponding author(s): Jackie Norrie and Michael Dyer

Last updated by author(s): 2021-6-11

Reporting Summary

Nature Research wishes to improve the reproducibility of the work that we publish. This form provides structure for consistency and transparency in reporting. For further information on Nature Research policies, see our Editorial Policies and the Editorial Policy Checklist.

Statistics

For all statistical analyses, confirm that the following items are present in the figure legend, table legend, main text, or Methods section.

n/a	Confirmed	
☐	☒	The exact sample size (*n*) for each experimental group/condition, given as a discrete number and unit of measurement
☐	☒	A statement on whether measurements were taken from distinct samples or whether the same sample was measured repeatedly
☒	☐	The statistical test(s) used AND whether they are one- or two-sided<br>*Only common tests should be described solely by name; describe more complex techniques in the Methods section.*
☒	☐	A description of all covariates tested
☒	☐	A description of any assumptions or corrections, such as tests of normality and adjustment for multiple comparisons
☐	☒	A full description of the statistical parameters including central tendency (e.g. means) or other basic estimates (e.g. regression coefficient) AND variation (e.g. standard deviation) or associated estimates of uncertainty (e.g. confidence intervals)
☒	☐	For null hypothesis testing, the test statistic (e.g. *F*, *t*, *r*) with confidence intervals, effect sizes, degrees of freedom and *P* value noted<br>*Give P values as exact values whenever suitable.*
☒	☐	For Bayesian analysis, information on the choice of priors and Markov chain Monte Carlo settings
☒	☐	For hierarchical and complex designs, identification of the appropriate level for tests and full reporting of outcomes
☒	☐	Estimates of effect sizes (e.g. Cohen's *d*, Pearson's *r*), indicating how they were calculated

Our web collection on statistics for biologists contains articles on many of the points above.

Software and code

Policy information about availability of computer code

Data collection

No code was used for data collection

Data analysis

RNA-sequencing Analysis
The paired end sequencing reads were subjected to mouse read cleansing with “bbsplit” (https://sourceforge.net/projects/bbmap/) if the sample was derived from xenografts. The adapters in sequencing reads were trimmed with “trim_galore” (v0.4.4, https://www.bioinformatics.babraham.ac.uk/projects/trim_galore/, -q 20 –phred 33 --paired). The trimmed sequencing reads were mapped with STAR2 to human genome GRCh38. The expected gene counts calculated using RSEM5 for each sample were compiled to one gene count matrix. Only genes annotated as level 1 or 2 by GENCODE (v31) were kept in the downstream analysis. In addition, only genes with count per million (CPM) more than 0.5 in at least one sample were kept. The normalization factor for each sample was calculated using “calcNormFactors” in the “edgeR” package (v3.26.8), and gene expression values were transformed and normalized using voom in the “limma” package (v3.40.6) in R. The normalized expression values were then used to perform principle component analysis (PCA) using the “prcomp” function in R. The loadings of PC1 and PC2 were used to generate the PCA plot.

The variance of the normalized gene expression value of each gene across all samples were calculated, and the top 500 most variable genes were used for the unsupervised clustering analysis. The clustering was performed using the “complete linkage” method (“hclust” function in R) based on Euclidean distances. The heatmap ordered by hierarchical clustering results was generated using the “ComplexHeatmap” package (v2.0.0) in R.

Methylation Array Analysis
Unsupervised hierarchical clustering (Euclidean Distanceand “Ward.D2” linkage) of DNA methylation profiling was performed based on the 5000 mostvariable methylation probes across all samples (or subset of samples), which were selected by variance of the beta values. For dimensionality reduction and visualization, PCA was performed in the initial steps using top 5000 most variable probes and first 50 dimensions were retained to run tSNE with perplexity values in the range (5-20) and 5000 iterations (“Rtsne” package, version 0.15, https://github.com/

jkrijthe/Rtsne). Copy number variation (CNV) analysis from methylation array data was performed using the "Conumee" package (version 1.16.0). Most differentially methylated regions were detected with DMRcate (version 1.18.0).

Whole Genome Sequence Analysis
The paired end sequencing reads were mapped with bwa. In addition, we used an ensemble approach to call somatic mutations (SNV/indels) with multiple published tools, including Mutect2 (v4.1.2.0), SomaticSniper (v1.0.5.0), VarScan2 (v2.4.3), MuSE (v1.0rc) and Strelka2 (v2.9.10). The consensus calls by at least two callers were considered as confident mutations. The consensus call sets were further manually reviewed for the read depth, mapping quality, and strand bias to remove additional artifacts. In terms of somatic copy number alternations (SCNA), in addition to CONSERTING, which is described previously, they were also determined by CNVkit (Talevich E. et all, 2016) and cn.Mops.
For somatic structural variants, five SV callers were implemented in the workflow for SV calling, including Delly (v0.8.2), Lumpy (v0.2.13), Manta (v1.5.0), Gridss (2.5.0) and novoBreak (v1.1). The SV calls passing the default quality filters of each caller were merged using SURVIVOR and genotyped by SVtyper. The intersected call sets were manually reviewed for the supporting soft-clipped and discordant read counts at both ends of a putative SV site using IGV.

Single cell RNA-seq analysis
Approximately 10,000 cells from each sample were taken and loaded onto the 10x chromium controller for single cell RNA sequencing analysis which was completed according to the 10x genomics protocol. Barcoded RNA was sequenced according to 10x Genomics protocol on an illumina HiSeq 2500 or 4000.Cell type recognition was determined using SingleR (v1.0.1)60 and copy number variation (CNVs) were identified using inferCNV (v1.2.1, inferCNV of the Trinity CTAT Project. https://github.com/broadinstitute/inferCNV).
In human fetal retina datasets, only cells with more than 500 genes and less than 3000 genes expressed and with less than 5% of mitochondrial reads are retained for analysis. The retained data were normalized and scaled using the SCTranform method in Seurat 3. Dimensionality reduction and clustering were also performed using Seurat functions. A list of genes indicating S and G2M phases of the cell cycle, compiled from the cell cycle genes provided in Seurat and in the G2M human genes provided in Aldiri et. al., were used to identify proliferating progenitor cells. Only clusters with enriched expression of at least three of those genes (adjusted p-value < 0.05, average logFC > 0.5, and percent of cells in the cluster expressing the genes is 1.5 folds higher than that of the rest of cells) were assigned as progenitor cells.
Single cell RNA-seq reads from tumors and normal retina samples were aligned using the cell ranger pipeline (v3.0.2) to the hg19 reference data (v3.0.0). Aligned data were processed using Seurat 3. Specifically, only cells with more than 400 genes and less than 7000 genes expressed and with less than 10% of mitochondrial reads are kept for downstream analyses. All cells in normal retina samples were then merged into one dataset. Cells in normal retina and all tumor datasets were scored for their cell cycle phase using the CellCycleScoring function in Seurat 3, based on the combined list of cell cycle genes provided in Seurat and in the G2M human genes
provided in Aldiri et. al. Normalization, scaling, dimensionality reduction, and clustering of normal retina and individual tumor datasets were performed as described above, but with cell cycle effects regressed out. Cell types in the normal retina dataset were identified based on enriched expressions of signature genes.
The identified progenitor cells across all ages in the fetal retina dataset were then combined with the normal adult retina dataset to serve as a reference for identification of cell types in tumor samples. Label transfer was performed using the default pipeline in Seurat 3 with FindTransferAnchors and TransferData functions.

RNA velocity analysis
Counts of unspliced and spliced reads from each tumor sample were derived using the velocyto command line tool, and reads mapped to multiple locus or mapped inside repeat regions (derived from UCSC genome browser) were discarded. The generated loom files were then analyzed using scVelo with a generalized dynamical model. Briefly, only genes with at least 10 counts for spliced and unspliced RNAs were kept for velocity analysis. Normalization, modeling of transcriptional dynamics, and estimation of RNA velocities were preformed using default parameters in scVelo. For visualization, single cell velocities were projected onto the precomputed umap embedding from Seurat.

Flow Cytometry Analysis
Flow cytometry data was analyzed using FACSDiva 8.0.1

For manuscripts utilizing custom algorithms or software that are central to the research but not yet described in published literature, software must be made available to editors and reviewers. We strongly encourage code deposition in a community repository (e.g. GitHub). See the Nature Research guidelines for submitting code & software for further information.

Data

Policy information about availability of data

All manuscripts must include a data availability statement. This statement should provide the following information, where applicable:
- Accession codes, unique identifiers, or web links for publicly available datasets
- A list of figures that have associated raw data
- A description of any restrictions on data availability

All raw genomic data from Figures 3, 4, and 5 will be made publicly available on GEO database under accession code GSE174200, GSE174201, and GSE174202. SIngle cell sequencing data is available in a Cloud-based viewer (https://pecan.stjude.cloud/static/rbsinglecell).

Field-specific reporting

Please select the one below that is the best fit for your research. If you are not sure, read the appropriate sections before making your selection.

☒ Life sciences ☐ Behavioural & social sciences ☐ Ecological, evolutionary & environmental sciences

For a reference copy of the document with all sections, see nature.com/documents/nr-reporting-summary-flat.pdf

Life sciences study design

All studies must disclose on these points even when the disclosure is negative.

Sample size	Sample size was determined by the number of tumors generated in the course of the study using 15 patient derived iPSC lines.
Data exclusions	No data was excluded
Replication	Validation of the RB mutation retention of the iPSC lines were completed on 3 independent clones.<br>We used 15 independent patient derived induced pluripotent stem cell lines to show retinoblastoma could be generated from iPSC derived retinal organoids. Retinal differentiation of each line was done in replicate by 2 different technicians.<br>Analysis of tumors by Taqman qRTPCR was completed on the tumors (n=14). 12 had markers of retinoblastoma, 2 (from the same iPSC line) were not retinoblastoma.
Randomization	Randomization was not completed for this study, a variety of RB mutations were used to accurately reflect the mutations found in patients with germ line mutations in RB.
Blinding	Blinding was not relevant to this study, there were no data that would influence the outcome of tumor formation from the iPSCs.

Reporting for specific materials, systems and methods

We require information from authors about some types of materials, experimental systems and methods used in many studies. Here, indicate whether each material, system or method listed is relevant to your study. If you are not sure if a list item applies to your research, read the appropriate section before selecting a response.

Materials & experimental systems

n/a	Involved in the study	
☐	☒	Antibodies
☐	☒	Eukaryotic cell lines
☒	☐	Palaeontology and archaeology
☐	☒	Animals and other organisms
☐	☒	Human research participants
☐	☒	Clinical data
☒	☐	Dual use research of concern

Methods

n/a	Involved in the study	
☒	☐	ChIP-seq
☐	☒	Flow cytometry
☒	☐	MRI-based neuroimaging

Antibodies

Antibodies used	PAX6 DSHB AB_528427 Mouse 1:100<br>Recoverin Millipore AB5585 Rabbit 1:5000<br>VSX2 Exalpha Biologics Inc. X1180P Sheep 1:200<br>OTX2 Santa Cruz sc-30659 Goat 1:200<br>OCT3/4 BD Biosciences BD 611202 Mouse 1:500
Validation	The Pax6, Recoverin, Vsx2, and Otx2 antibodies have been routinely used to identify retinal cells in retina. Here we used them to identify retinal cell types in organoids, using retina as a control.Hiler D, Chen X, Hazen J, Kupriyanov S, Carroll PA, Qu C, Xu B, Johnson D, Griffiths L, Frase S, Rodriguez AR, Martin G, Zhang J, Jeon J, Fan Y, Finkelstein D, Eisenman RN, Baldwin K, Dyer MA. Quantification of Retinogenesis in 3D Cultures Reveals Epigenetic Memory and Higher Efficiency in iPSCs Derived from Rod Photoreceptors. Cell Stem Cell. 2015 Jul 2;17(1):101-15. doi: 10.1016/j.stem.2015.05.015. PMID: 26140606; PMCID: PMC4547539.<br><br>Oct3/4 has been used to identify pluripotent stem cells. Watanabe K, Ueno M, Kamiya D, Nishiyama A, Matsumura M, Wataya T, Takahashi JB, Nishikawa S, Nishikawa S, Muguruma K, Sasai Y. A ROCK inhibitor permits survival of dissociated human embryonic stem cells. Nat Biotechnol. 2007 Jun;25(6):681-6. doi: 10.1038/nbt1310. Epub 2007 May 27. PMID: 17529971.

Eukaryotic cell lines

Policy information about cell lines

Cell line source(s)	H9 (WA09) WiCell
Authentication	Powerplex was completed to authenticate the lines
Mycoplasma contamination	All cell lines tested negative for mycoplasma.

Commonly misidentified lines (See ICLAC register)	No commonly misidentified lines were used in this study

Animals and other organisms

Policy information about studies involving animals; ARRIVE guidelines recommended for reporting animal research

Laboratory animals	All animal procedures and protocols were approved by the St. Jude Laboratory Animal Care and Use Committee. All studies conform to federal and local regulatory standards. Female C57BL/6 scid mice were purchased from Jackson Laboratories (strain code 001913). Mice were housed on ventilated racks on a standard 12-hour light-dark cycle.
Wild animals	This study did not involve wild animals
Field-collected samples	This study did not involve field-collected samples
Ethics oversight	The St. Jude Animal Care and Use Committee and the The St. Jude Children's Research Hospital Institutional Review Board approved and provided guidance on the study protocol.

Note that full information on the approval of the study protocol must also be provided in the manuscript.

Human research participants

Policy information about studies involving human research participants

Population characteristics	Eligibility criteria included a family history of retinoblastoma with an identified germline RB1 mutation, diagnosis of bilateral retinoblastoma, or diagnosis of unilateral retinoblastoma with germline RB1 mutation, MYCN amplification, or 13q deletion identified. Participants were male and female, ages 4 months to 36 years.
Recruitment	Written informed consent was obtained from each participant or participant's parent/guardian. Eligibility criteria included a family history of retinoblastoma with an identified germline RB1 mutation, diagnosis of bilateral retinoblastoma, or diagnosis of unilateral retinoblastoma with germline RB1 mutation, MYCN amplification, or 13q deletion identified. Participants with a variety of RB1 mutations were chosen to ensure a specific mutation would not effect the iPSC creation, retinal organoid creation, or tumor formation.
Ethics oversight	RETCELL (NCT02193724), a protocol to establish the feasibility, validation and differentiation of induced pluripotent stem cells produced from patients with heritable retinoblastoma, was approved by the St. Jude Children's Research Hospital Institutional Review Board and open to accrual in July 2014.

Note that full information on the approval of the study protocol must also be provided in the manuscript.

Clinical data

Policy information about clinical studies
All manuscripts should comply with the ICMJEguidelines for publication of clinical research and a completedCONSORT checklist must be included with all submissions.

Clinical trial registration	NCT02193724
Study protocol	The goal of this study is to determine if human RB1-deficient induced pluripotent stem cells (iPSCs) can produce retina, and, furthermore, can give rise to retinoblastoma in culture. This unique opportunity to study the initiation of retinoblastoma in the developing retina will shed light on the cell of origin for retinoblastoma and allow the investigators to study the earliest molecular and cellular events in retinoblastoma tumorigenesis.
Data collection	Participants identified with heritable retinoblastoma will undergo a skin biopsy or blood draw to collect cells for processing and analysis.
Outcomes	Skin biopsy or peripheral blood mononuclear cells will be collected from eligible, consenting participants and shipped directly to the University of Wisconsin for processing. All samples will be returned to the St. Jude investigator within two months of reprogramming for further analysis.

Flow Cytometry

Plots

Confirm that:

☒ The axis labels state the marker and fluorochrome used (e.g. CD4-FITC).

☒ The axis scales are clearly visible. Include numbers along axes only for bottom left plot of group (a 'group' is an analysis of identical markers).

☒ All plots are contour plots with outliers or pseudocolor plots.

☒ A numerical value for number of cells or percentage (with statistics) is provided.

Methodology

Sample preparation	iPSCs were dissociated using Accutase. PE conjugated anti-TRA-1-81(BD, #560885) or FITC conjugated anti SSEA4 (BD, #560126)
Instrument	BD Fusion
Software	FACSDiva 8.0.1
Cell population abundance	iPSCs were confirmed to be TRA-1-81 and SSEA4 positive
Gating strategy	Gates were set using unstained and single stained cells.

☒ Tick this box to confirm that a figure exemplifying the gating strategy is provided in the Supplementary Information.
